# Excess body weight, diabetes and cancer: epidemiologic evidence implicating hormonal and metabolic mechanisms

**DOI:** 10.1186/1753-6561-6-S3-O12

**Published:** 2012-06-01

**Authors:** Rudolf Kaaks

**Affiliations:** 1 Division of Cancer Epidemiology; German Cancer Research Center (DKFZ), Heidelberg, Germany

## 

Epidemiological observations increasingly implicate nutritional energy balance as a key risk factor for cancer development. Excess body weight is associated with increased risks of cancers of the endometrium, breast (postmenopausal women), kidney (renal cell tumours), colon, pancreas and oesophagus (adenocarcinomas), and is also a well-documented risk factor for high-grade prostate cancer. By contrast, regular physical activity reduces the risks of breast and colorectal cancers and potentially other tumour types, and overall, excess weight and lack of physical activity have been estimated to potentially account for one quarter to half of the occurrence of the most frequent tumour types in affluent, industrialized societies. Animal experiments have shown uniformly protective effects of dietary energy restriction against tumor development.

Physiologic mechanisms that are thought to account for these effects of nutritional energy balance on cancer risks include changes in the metabolism of endogenous hormones, growth factors and inflammation factors, as well as in energy and nutrient status at the level of single cells. Together, these physiologic changes may stimulate cell growth and proliferation, inhibit apoptosis, and favour the occurrence of genetic mutations through increased oxidative stress. The key mechanisms that underlie these relationships of nutritional energy balance with cancer development may strongly depend, however, on tumour type.

Prospective cohort studies have shown relationships of risks of various cancer types with blood levels of glucose and insulin, and insulin-like growth factor-l. Furthermore, increased levels of circulating androgens and estrogens and reduced levels of progesterone are strongly implicated especially in the development of cancers of the endometrium and breast, among women with excess weight. The implication of specific hormonal or metabolic factors in cancer development is further increased by a growing body of evidence from human intervention studies – e.g. using selective estrogen receptor modulators [SERMS] and aromatase inhibitors – and epidemiological studies on the effects of specific hormonal medications (e.g., oral contraceptives and postmenopausal hormone replacement therapy; specific anti-diabetic drugs). Finally, several studies have shown increased risks of cancer, e.g. of the colorectum and endometrium, among subjects with higher than average serum levels of inflammation factors such as C-reactive protein, TNF-alpha or various interleukins.

According to present estimates, excess weight and lack of physical activity are likely the most important risk factors after smoking for cancer occurrence overall in western countries, and better knowledge of the true magnitude of the problem and of physiologic mechanisms involved can help define guidelines for primary prevention. At the same time, the knowledge gained about the hormonal and other physiologic mechanisms that link nutritional energy balance to cancer increasingly is also informing translational research into cancer preventive and curative treatments.

